# Hyper-IgE Syndrome with STAT3 Mutation: A Case Report in Mainland China

**DOI:** 10.1155/2010/289873

**Published:** 2010-05-17

**Authors:** Lixin Xie, Xiaoxiang Hu, Yang Li, Weihua Zhang, Liang'an Chen

**Affiliations:** ^1^Department of Respiratory Medicine, Chinese PLA General Hospital, 28th Fuxing Road, Beijing 100853, China; ^2^State Key Laboratory for Agribiotechnology, China Agricultural University, Beijing 100094, China

## Abstract

Hyper-immunoglobulin E syndromes (HIES) including compound primary immunodeficiency and nonimmunological abnormalities are characterized by extremely high serum IgE levels, eosinophilia, eczema, susceptibility to infections, distinctive facial appearance, retention of deciduous teeth, cyst-forming pneumonias, and skeletal abnormalities. Itis reported that some cases of familial HIES are relative to autosomal dominant or recessive inheritance, but most cases are sporadic, and result from mutations in the human signal transducer and activator of transcription 3 (STAT3) gene. In this paper, we firstly report a young man diagnosed of Hyper-IgE syndrome with STAT3 mutation in Mainland China, and investigate the autosomal dominant trait of his family members.

## 1. Introduction

The hyper-IgE syndromes (HIES) are rare primary immune deficiencies characterized by elevated serum IgE, dermatitis, and recurrent skin and lung infections. There are two forms of HIES: a dominant form caused by mutations in STAT3, and a recessive form, for which a genetic cause is unclear. These two different syndromes have distinct presentations, courses, and outcomes and share very little in terms of pathogenesis other than the IgE elevation. In this paper, we firstly report a young man diagnosed of Hyper-IgE syndrome with STAT3 mutation in Mainland China, and investigate the autosomal dominant trait of his family members.

 Our patient, a 20-year-old man in mainland China, was suffering from eczema, lung cyst, skeletal and dental abnormalities, and so forth, which are the characteristics of type 1 HIES. He has extremely high serum IgE level is 200 times than that of normal person. Then, we sequenced the STAT3 gene by complementary DNA (cDNA) and genomic DNA, and we found a mutation locus, in which his parents and sister are normal.

## 2. Patient and Diagnosis

A 20-year-old man was found to presented with over ten-years history of cough with yellow-colored sputum and one year history of bloody sputum. He also reported universal boils on the face and limbs and recurrent pneumonias. In 1998, he received right upper lung cyst surgical therapy. His medical history was significant for eczema since newborn period and recurrent pustular and eczematoid rashes on the face and scalp in the childhood. Several primary teeth arrachement surgeries were performed in 13 years old on account of failure of the primary teeth to exfoliate. 

**Figure 1:**
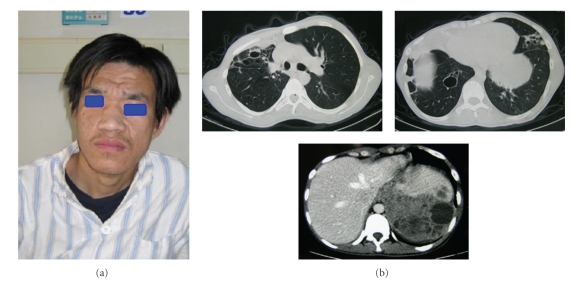
Symptoms. (a) The patient with broad nose, deep-set-eyes, a prominent forehead, and a rough facial skin with exaggerated pore size. (b) Multiple cysts in the left upper abdomen.

 On physical examination, the vital signs were normal, clubbed fingers and toes; the characteristic facial appearance was noted with broad nose, deep-set eyes with a prominent forehead (Figure 1(a)), and a rough facial skin with exaggerated pore size. Flat chest, scattered rash scars were showed on the chest skin. Bilateral fine crackles were audible in the lower lung, decreased breath sounds at the right base, and scattered expiratory wheeze bilaterally. Abdominal examination revealed moderate left middle abdominal tenderness.

The leukocyte count was 11,350 cells/L (62% neutrophils, 19% lymphocytes, and 9% eosinophils). The hemoglobin concentration was 104 g/L, the red blood cell count 3.82 × 10^12^/L, and the platelets count was 354 × 10^9^/L. The erythrocyte sedimentation rate (ESR) was 87 mm/h. The C-reactive protein (CRP) was 7.15 mg/dL. The findings of further laboratory workup included the following: serum IgA, 135.0 mg/dL (reference range, 70 to 400 mg/dL); serum IgG, 2,560.0 mg/dL (reference range, 700 to 1600 mg/dL); and serum IgE, 37,700.0 IU/mL (reference range, 0 to 100 IU/mL). IgM concentrations (subclasses included) were within the normal range. CD4/CD8 count was normal. The findings of an enzymelinked immunosorbent assay for HIV antibody and an antineutrophil antibody screen were negative. 

 A chest CT scan showed extensive consolidation and cystic changes in the right lung and patchy infiltration and cystic changes in the left lower lung. An abdominal CT scan revealed a big lower density mass measured 10.6 cm × 9.5 cm with multiple cysts in the left upper abdomen (Figure 1(b)). Bronchoscope examination found multimucous sputum in the tracheal and right and left bronchus. Percutaneous abdominal mass puncture and drainage guided by ultrasonography were performed, and laboratory examination of drainage liquid reported purulent fluid with a great amount of leukocytes. Cultivation of bacteria both in abdominal abscess and bronchial alveolar lavage fluid (BALF) revealed staphylococcus aureus (Methicillin sensitive staphylococcus aureus, MSSA). 

Based on these findings, we made the diagnosis of Hyper-IgE syndrome (HIES). According to these characteristics, we confirmed it the type 1 HIES. (This study was approved by the patient and informed consent was obtained from the families).

## 3. Material and Method

### 3.1. Mutational Analysis

EDTA blood was obtained and genomic DNA was isolated by using standard methods. All 24 exons and exon/intron boundaries were separately amplified by PCR. (The primers were designed according to GenBank NG_007370, the mutation in Exon 15 was amplified with 15F-5′-GATGGAGTTTTGCTGTGCTG-3′ and 15R-5′-AGATG GGATGCCAAGGATTT-3′).

### 3.2. Total RNA Extraction and cDNA Preparation

Total RNA was prepared from leucocyte of whole blood samples of the patient and his families using the QIAZOL Lysis Reagent (RNeasy Lipid Tissue) isolation method according to the manufacturer's protocols (Qiagen, Valencia, CA, USA). Total RNA was isolated from by an RNeasy mini kit (Qiagen, Valencia, CA, USA) according to the manufacturers' instructions. About 1 *μ*g RNA was reverse transcribed into single-strand cDNA using oligo(dT) 18-mer primers and M-MLV Reverse Transcriptase (Invitrogen, Karlsruhe, Germany).

### 3.3. Quantitative Real-Time RT-PCR

Real-time RT-PCR was performed on a fluorescence thermal cycler (ABI Prism 7900 HT Sequence Detection System, Applied Biosystems). A standard two-step procedure was applied. RNAs were reverse transcribed into single strand cDNAs using oligo dT primers and M-MLV reverse transcriptase (Invitrogen, Karlsruhe, Germany). Real-time RT-PCR was performed in 15-*μ*L reaction mixtures consisting of cDNA, 0.5 *μ*M specific primer sets for each target gene, and SYBR Green PCR Master Mix (Applied Biosystems, UK). Conditions were: 50°C for 2 minutes, 95°C for 10 minutes, followed by 40 repetitive cycles of 95°C for 15 seconds, and 60°C for 1 minute. Details of melting curve analysis and relative standard curve generation were described in Supplemental Materials and Methods. *β*-actin was used as internal controls to normalize for initial RNA input and this gene was found to display remarkably stable expression levels across experimental treatments. (The primers of stat3 gene for RT-PCR were F: 5′-CAGTCCGTGGAACCATACACA-3′, R: 5′-GACCAGTGGAGA CACCAGGATA-3′; the primers of *β*-actin for RT-PCR were F: 5′-AAGATCATTGCTCCTCCTGAGC-3′, R: 5′-TCCTGCTTGCTGATCCACATC-3′).

### 3.4. Analysis of Allelic Expression of STAT3 Gene

Pyrosequencing technology on the PyroMark ID instrument was used to analyze allelic expression of stat3 gene at the mutational locus. Amplifying the dsDNA by PCR with one biotinylated primer (R-pyro: 5′-CACGACGTTGTAAAACGACGGA GTGGGTCTCT-3′) and one unlabelled primer (F-pyro: 5′-CCGAGCCAATTGTGAT GC-3′); immobilizing the DNA samples, typically 2 pmol PCR product, 109 bp in length (CCGAGCCAATTGTGATGCTTCCCTGATTGTGACTGAGGAGCTG  C[A/C]CCTGATCACCTTTGAGACCGAGGTGTATCACCAAGGCCTCAAGATTGACCTAGAGACCCACTCC), to Streptavidin Sepharose beads; separating the DNA strands; dispensing the ssDNA samples into the wells of the PSQ 96 Plate Low; annealing the samples to a sequencing primer (Seq-pyro: 5′-TGACTGAGGAGCTG C-3′) were carried out. 

## 4. Results

### 4.1. The Identified Mutation in the STAT3 Gene

**Figure 2:**
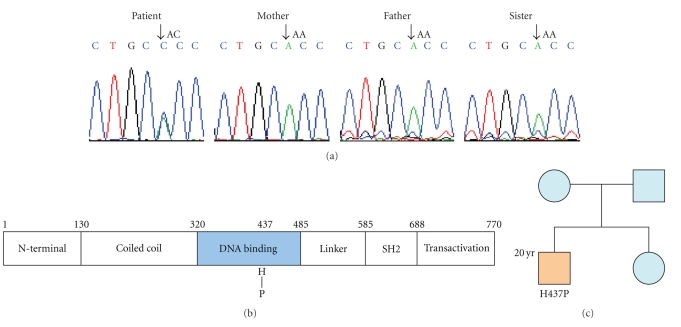
Sequencing results of STAT3 cDNA. (a) Heterozygous mutation in STAT3 genomic sequence of the patient. (b) Schematic of STAT3 amino acid structure and the identified mutation, H437P (1310A → C) in the DNA binding domain. (c) Pedigree of this family affected by the Hyper-IgE syndrome, the mutation was de novo, not inheritance.

Several previous studies have showed mutations in the STAT3 gene on chromosome 17q21 as major causes of AD and sporadic HIES [1, 2]. We thus sequenced the regulatory region, the coding exons, and the intron-exon junctions of the STAT3 gene in the patient and his families. A heterozygous mutation H437P (1310A → C) occurred only in the patient who had extremely high IgE level. This mutation seemed to be de novo since none of the parents and the sibling of the patients carried this mutation, (Figure 2) with Mildly to moderately elevated IgE levels for his mother (162 IU/mL) and his younger sister (887 IU/mL).

### 4.2. The Gene Express Analysis of STAT3 by Real-Time PCR

**Figure 3:**
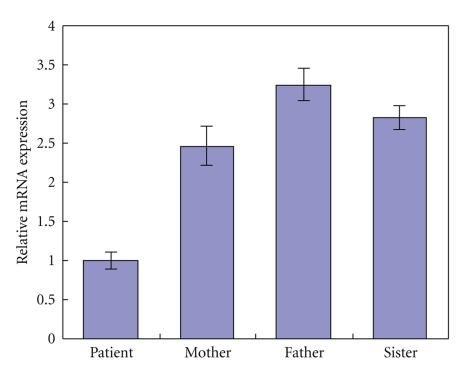
Relative STAT3 mRNA expression levels in the family. The expression of the patient is twice times lower than his families control.

The mRNA expression of STAT3 gene in the leucocyte from whole blood samples of the patient and his families control was examined by RT-PCR, using a pair of specific primers amplifying a 111-bp amplicon of this gene. The house-keeping gene *β*-actin was used as an internal control for normalization. A relatively reduction level of expression of STAT3 gene can be observed in the patient (Figure 3).

### 4.3. The Allele Expression Quantified by cDNA Pyro-Sequence

**Figure 4:**
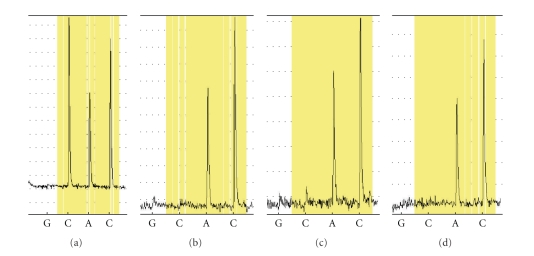
Pyrosequencing results (a) 38.9% C and 61.1% A at the mutational point of the patient. (b)–(d) 100.0% A and 0.0% C at the corresponding point of his parents and sister.

To study this mutated allele expression more directly, we quantified the relative expression of alleles in leucocyte from whole blood samples of the patient and his families control using the 1310A → C SNPs located in the Exon 15 of STAT3 gene. cDNA sequences from patient samples revealed expression of both alleles (38.9% C and 61.1% A through the exactly calculation) indicating the mutated transcription of STAT3 gene present in the patient. The association between the expression levels implies that the 1310A → C SNPs is the causative mutation for this dominant case (Figure 4).

## 5. Discussion

In 1966, the syndrome was first described as “Job's syndrome” by Davis et al. in two girls suffering from recurrent “cold” staphylococcal abscesses, pneumonia, and neonatal-onset eczematoil rash, referring to the Biblical Job, who was “smote with sore boils” [3]. Then the disease was reported as hyper-IgE syndrome by Buckley et al. in 1972 because they found that these symptoms were associated with exceptionally high serum concentrations of IgE [4].

 HIES is a rare immunodeficiency syndrome of which the exact pathogenesis is still unknown. There is no specific clinical and laboratory test for confirming. Several symptoms such as elevated IgE levels and eosinophilia might also be found in other immunodeficiency syndromes [5]. Therefore, we must synthetically analyze the medical history, appearance and skin characteristics, visceral abnormalities, and necessary laboratory study findings including cytokines and immunoglobulins levels. Our patient's pathogenesis was not working out until all the abovementioned aspects were examined. And all the symptoms indicated that he is a patient with hyper-IgE syndrome.

 The diagnosis of HIES is difficult to be confirmed in that both immunologic and somatic features need to be identified prior to genetic testing. There are two forms of HIES [6]. They have different pathogenesis, processes, and outcomes, and the only common ground is the IgE elevation, with values reaching >2000 IU (normal <200 IU) [7]. The type 1 HIES, a dominant form caused by hypomorphic mutations in STAT3, is a disease of multiorgan dysfunction. Besides eczema and recurrent staphylococcal infections in skin and lung, these patients suffer from abnormalities in vessels, connective tissue, and skeleton [8]. STAT3 (signal transducer and activator of transcription 3) is located on human chromosome 17q21, which was reported to contain a disease locus for familial autosomal dominant (AD)-HIES [7]. It is a transcription factor, which binds to the STAT3-responsive elements in the promoters of various genes and plays a critical role in responses to many cytokines, in which, IL-17 produced by T_H_17 cell is protective in the host defence against extracellular bacteria [9], and IL-22 stimulates cells in the skin and respiratory systems to produce *β*-defensins through STAT3 activation [10]. Therefore, the HIES aetiology might be directly and indirectly linked to STAT3. In other words, a human deficiency in STAT3 is a major cause of sporadic and familial HIES. The type 2 HIES is autosomal recessive (AR) syndrome [11]. The patients with type 2 HIES did not show any skeletal and dental abnormalities, and had no pulmonary cyst, but most of them suffered from viral infections such as chronic refractory molluscum contagiosum and herpes simplex virus infections, which were not identified in type 1 HIES. The genetic origin for a subpopulation of type 2 HIES is a null mutation of tyrosine kinase 2 (Tyk2) [12].

 For the past few years, the research in the etiology of HIES has got some achievements, especially with the development of molecular biology. But it is not completely clear to us. Until now, it is generally believed that STAT3 mutations act in a dominant negative manner to cause of autosomal dominant HIES [13]. And Tyk2 deficiency acts in a recessive manner to cause one of the cases of AR-HIES, although other genomic loci may also be involved [14]. Most STAT3 mutations are restricted to the DNA-binding or SH2 domains [15], and might concern the protein level, phosphorylation, and nuclear localization. In our research, we found a heterozygous mutation in the DNA-binding region of STAT3 gene, it could be one of the essential causes of the disease. Furthermore, may be his lower STAT3 protein expression level is a result of the mutation. However, its concrete effect still remains to be studied, for instance, when the mutation happens, how the STAT3 protein structure changes, and how the underlying mechanisms and passages make mistakes. They all need us to explore deeply.

## 6. Conclusion

These new and evolving genetic and immunologic understandings probably eventually lead to more effective disease-specific treatment for patients, including stem cell transplantations and gene-targeted therapies.
